# On the Mississippi

**Published:** 1885-02

**Authors:** 


### ON THE MISSISSIPPI.

Of the scenery of the lower Mississippi there is not much to be said; it is absolutely flat on either bank. There is the novelty to northern eyes of gliding between fields of waving sugar cane, alternated with acres of orange groves, as long as we like in the State of Louisiana; but once that State is passed and we are traveling through Tennessee and Mississippi States there is nothing to redeem the general ugliness. The boat stops at numberless small landings with strange names, to take or put off passengers or freight—a bale or two of cotton, sometimes sugar. Occasionally these little settlements are verdant, prosperous-looking places, with cheery people to watch the boat coming in; but more often they are such as remind one of Dickens' "Eden;" the yellow ague-worn faces, the weary, listless air, telling of the fatal malaria that dwells in these water-soaked lands. Every now and again some spot of interest in connection with the rebellion comes in view—Baton Rogue, Natchez, Vicksburg, all large, handsome cities —but to specify the points of historic interest would carry us beyond the limits of this paper. Dickens has told us of the sluggish, filthy river, whose turbid and lonesome waters brought disease and death to the poor settler at Eden. But, though he has in no way exaggerated the foul appearance of the water and believed in calling it filthy, he was correct; it is probably more free from actual filth than many a river of bright, running water. Its appearance is as dirty a hundred miles from a large city as in its vicinity, which would indicate that its dark appearance is not due to sewage; far fewer factories discharge their contents into it during its whole length than into the Thames, and as all the population that dwell on its banks is probably less than that of London, while the volume of water is a great many times more than in the Thames, whatever is foul must be more largely diluted. Yet the water is disgusting in appearance, being like coffee to which a very little milk has been added; it is nearly opaque, and even when filtered it is very uninviting in appearance: yet those who drink it protest that it is wholesome, and some account for its appearance by saying it is dyed by the bark of trees from the forrest through which it passes, which gives it the tan colored appearance; and in some parts of the stream huge quantities of bark are found floating in it. A planter on the stream told us his men had caught at least 40 cords of wood that spring which had floated down below New Orleans, and that the planters generally obtained their supply in this way.

